# Long Term Progression-Free Survival in a Patient with Locally Advanced Prostate Cancer under Low Dose Intermittent Androgen Deprivation Therapy with Bicalutamide Only

**DOI:** 10.1155/2015/928787

**Published:** 2015-03-26

**Authors:** Stefan Latz, Christian Fisang, Wolfram Ebert, Stefan Orth, Dirk G. Engehausen, Stefan C. Müller, Ralf Anding

**Affiliations:** ^1^Department of Urology, University Hospital Bonn, Sigmund-Freud-Straße 25, 53127 Bonn, Germany; ^2^Department of Urology, Kreiskrankenhaus Luedenscheid, Paulmannshöher Straße 14, 58515 Luedenscheid, Germany; ^3^Department of Urology, Klinikum-Westfalen GmbH, Am Knappschaftskrankenhaus 1, 44309 Dortmund, Germany; ^4^Department of Urology, University Hospital Erlangen, Krankenhausstraße 12, 91054 Erlangen, Germany; ^5^Sinntal Hospital Bad Brückenau, Hospital of the Deutsche Rentenversicherung Nordbayern, Wernarzer Straße 12, 97769 Bad Brückenau, Germany

## Abstract

Androgen deprivation is a common treatment option in patients with locally advanced or metastatic prostate cancer. No case of long term treatment with an intermittent approach with only low dose bicalutamide (50 mg daily) has been described yet. We report a 60-year-old patient, initially presenting with a PSA elevation of 19.2 ng/mL in 1996. After diagnosis of well to moderately differentiated prostate cancer by transrectal biopsy, the patient underwent an open radical prostatectomy. Final diagnosis was adenocarcinoma of the prostate, classified as pT3a, pR1, pV0, and pL1. Adjuvant intermittent androgen deprivation therapy with flutamide 250 mg was applied, which was changed to bicalutamide 50 mg once daily when it became available in 2001. Six on-phases were performed and PSA values never exceeded 20 ng/mL. The patient did not experience any serious side effects. To date, there are no clinical or radiological signs of progression. Current PSA value is 3.5 ng/mL.

## 1. Introduction

Androgen deprivation has been established in the treatment of advanced prostate cancer for over 50 years. However, continuous androgen deprivation goes along with many short and long term side effects. Tunn was the first to describe intermittent endocrine therapy of prostate cancer. Treatment goal was an improvement of quality of life between phases of active treatment [1]. Prerequisite is a testosterone recovery during off-treatment periods. Tunn et al. found a testosterone recovery in 79.3% during the first off-treatment cycle with a median time to normalisation of 100 days [2].

Pros and cons of intermittent androgen deprivation are still under discussion. The intermittent approach failed in delaying castration resistance. There is also no overall survival benefit. However, trends for improvements in quality of life could be shown [3].

## 2. Case

In 1996, a 60-year-old man presented with an elevated PSA of 19.2 ng/mL. Digital rectal examination indicated a suspected tumor of the right prostatic lobe. The patient underwent a transrectal biopsy of the prostate. Histologically, a well to moderately differentiated adenocarcinoma of the prostate was found and a radical retropubic prostatectomy was performed. The preoperatively performed skeletal scintigraphy revealed no signs of bone metastases. A well to moderately differentiated adenocarcinoma of the prostate was confirmed, reaching the margin of the apical right prostatic lobe with infiltration of the prostatic capsule. Furthermore, focal lymphangiosis carcinomatosa was found. The tumor was finally classified as pT3a, pR1, pV0, pL1, and G2 (according to the 4th edition of the TNM system from 1992). At that time a Gleason grading was not conducted. Six weeks after surgery the PSA level was 0.00 ng/mL.

Due to the pathological findings an adjuvant androgen deprivation was recommended. However, the patient called for a therapy regime with as few side effects as possible and preservation of quality of life. For this reason also adjuvant local radiotherapy was refused by the patient. Only a prophylactic radiotherapy of the mamillae (cumulative dose 12 Gy) was conducted and androgen deprivation therapy with flutamide 250 mg (three times a day) started in July 1996. Hereunder, PSA levels remained stable around 1.5 ng/mL and androgen deprivation was stopped in November 1997.

**Figure 1:**
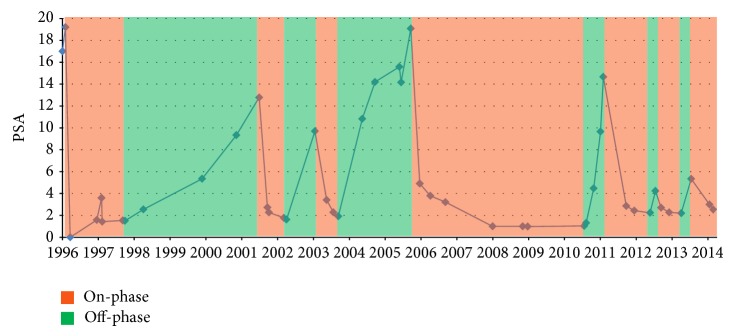
PSA course from 1996 to 2014. Colours illustrate therapy phases (green: off-phases 92/221 months; on-phases 129/221 months).

According to the PSA level course, a total of six on-phases were performed (combined 129/221 months in 18 years); see Figure 1. Trigger for reconvening on-phases was a PSA level between 10 and 20 ng/mL without a specific threshold. This was individually discussed with the patient at each visit. Therapy was changed to bicalutamide 50 mg once daily when it became available in 2001. Restaging examinations with bone scintigraphy in June 1997 and June 2004 as well as magnetic resonance imaging in August 1998 did not reveal any signs of progression. Regular clinical examinations including digital rectal examinations were also without pathological findings. PSA values never exceeded 20 ng/mL. Testosterone levels slowly increased over the years (December 2010: 26.9 nmol/L; September 2012: 37.6 nmol/L; August 2013: 41.4 nmol/L), but this is well known for bicalutamide monotherapy [4, 5]. Androgen resistance could not be observed as there was always immediate response to bicalutamide in on-phases with a repeated PSA decline below 3 ng/mL. In view of this positive response there was no need for considering salvage radiotherapy. During the course of therapy the patient never reported any serious side effects. The last PSA level was 3.5 ng/mL, and the patient is again in on-phase since November 2013.

## 3. Case Hypothesis and Rationale

We state that intermittent antiandrogen therapy with bicalutamide 50 mg once daily might be a treatment option for patients with locally advanced prostate cancer, calling for an adjuvant therapy with preferably little side effects. Requirements for the procedure are good patient compliance and frequent reevaluations with interim history, clinical examination including digital rectal examination, PSA testing, and radiological analysis when necessary.

## 4. Discussion

Bicalutamide binds to the ligand-binding domain (LBD; exons 5–8 on chromosome Xq11-12) of human androgen receptor (AR). In the wake of this bicalutamide inhibits androgen stimulated gene expression and cell growth, leading to tumor cell apoptosis [4]. It has only little effect on serum luteinizing hormone and testosterone as it acts as a pure and peripherally selective antiandrogen. Testosterone levels can even increase as the negative feedback on the release of luteinizing hormone is inhibited. Actually, we observed a continuous increase in testosterone levels in this case. However, adverse effects of the treatment are attributed to the simultaneously elevated estrogen levels [4, 5].

Carswell and Figgitt found that monotherapy of bicalutamide 150 mg once daily was as effective as medical or surgical castration in terms of overall survival in patients with locally advanced nonmetastatic prostate cancer [6]. No survival differences could be found in patients with locally advanced or metastatic prostate cancer given high-dose bicalutamide at 300, 450, and 600 mg per day [7].

Bicalutamide monotherapy is usually well tolerated and beneficial to quality of life with preservation of libido, sexual potency, and physical capacity, promoting patient compliance under therapy [4, 6].

To date, the role of immediate hormonal therapy after radical prostatectomy or external beam radiation is still under discussion. Early use of bicalutamide 150 mg once daily in patients with locally advanced prostate cancer may delay clinical progression of the disease. It can also be advantageous concerning overall survival in patients primarily treated with external beam radiation. However, no survival benefits could be found in patients with only localized disease [8].

Androgen deprivation goes along with many serious side effects like impairment of cognitive function, self-esteem, libido, and sexual function [9]. The intermittent approach reduces adverse effects but fails in delaying androgen resistance. There is also no survival benefit compared with the continuous approach. An intermittent therapy in metastatic disease is not recommended. Furthermore, patients with an inadequate PSA response are not qualified for an intermittent regime [3, 9].

Bicalutamide 50 mg once daily is approved for the use in combination therapy with a luteinizing hormone-releasing hormone (LHRH), and a dose of 150 mg is approved (in Germany) as adjuvant monotherapy in locally advanced PCA. Tyrrell et al. did not find any survival benefits of higher doses [7] that go along with potentially more adverse effects. Therefore, we state that androgen deprivation with bicalutamide 50 mg only might be justified as long as full patient compliance is assured. Beside the low PSA values over the years, we achieved a progression-free survival of 18 years with only low dose bicalutamide. In this long period of time no evidence of AR mutations was found potentially leading to local progression or metastases. However, when any signs of progression occur, change to standard therapy regimen is recommended. Just recently, a long term follow-up of 450 patients with clinically localized prostate cancer followed by active surveillance revealed a low rate of prostate cancer mortality [10]. For particular groups of patients less is sometimes more.
